# Exhaled 8-isoprostane as a prognostic marker in sarcoidosis. A short term follow-up

**DOI:** 10.1186/1471-2466-10-23

**Published:** 2010-04-27

**Authors:** Wojciech J Piotrowski, Zofia Kurmanowska, Adam Antczak, Jerzy Marczak, Paweł Górski

**Affiliations:** 1 Department of Pneumology and Allergy, Medical University of Lodz, Poland

## Abstract

**Background:**

8-Isoprostane (8-IP) is a marker of lipid peroxidation. Elevated concentrations have been reported in BAL fluid and exhaled breath condensate (EBC) in sarcoidosis (S). To validate the prognostic value of this marker we tested whether: 1. high initial EBC 8-IP predispose to more severe disease; 2. low initial concentrations increase a chance of early remission; 3. remissions are connected with the decrease of EBC 8-IP.

**Methods:**

40 patients (S) have been examined initially (V1) and after 8.5 ± 0.5 months (V2). EBC 8-IP concentrations were measured by ELISA. Chest X-ray, lung function test, serum ACE and Ca^2+ ^concentrations, 24 hrs Ca^2+^loss, abdominal ultrasonography, symptoms evaluation were performed.

**Results:**

We confirmed higher concentrations of 8-IP in EBC of patients with sarcoidosis (p = 0.001). Relative risk (RR) of persistence of disease at V2 when initial 8-IP was above 20 pg/mL was 1.04, and the frequency distributions estimated by χ^2 ^test were not significantly different. A chance (RR) of early complete remission when V1 8-IP was below DL, was 3.33 (p = 0.04 by χ^2 ^test). A significant decrease of 8-IP at V2 was observed only in patients who received treatment (p = 0.03), but not in those with spontaneous remission.

**Conclusions:**

We come to the conclusion, that low initial 8-IP may be a positive prognostic factor. A decrease of 8-IP in treated patients reflects a non-specific effect of treatment and is not related to mere regression of disease.

## Background

In more than 90% of patients suffering from sarcoidosis lung parenchyma and intrathoracic lymphnodes are involved. Lymphocytic inflammation with predominance of CD4+ cells and granuloma formation are the typical features of this disease. Remissions, usually spontaneous, are frequent, and in many patients the disease disappears within 6-12 months. Only about 5% of patients experience a progression towards lung fibrosis [1]. Today, one of most important unresolved questions is, which patients would benefit from treatment and what criteria or markers should be used for qualification [2].

Oxidative stress plays a causative role in idiopathic pulmonary fibrosis (IPF), a disease characterized by severe lung destruction and unresponsiveness to treatment [3]. Although alveolar inflammatory cells release high concentrations of superoxide anion in active sarcoidosis [4], secondary markers of oxidative stress, such as lipid peroxidation products or oxidized methionine are not elevated, contrary to IPF [5]. These observations established the opinion, that oxidative stress is not a crucial element in the pathogenesis of sarcoidosis. However, as only a minority of patients are in danger of irreversible lung fibrosis, it can not be excluded that oxidative stress with subsequent peroxidation of membrane lipids is also important in the pathogenesis of lung fibrosis in this selected group of sarcoid patients.

8-Isoprostane (8-IP) is a marker of oxidative stress. It is a prostaglandin-F_2α _isomer, produced *in vivo *by free radical-catalyzed peroxidation of arachidonic acid [6]. Elevated concentrations of 8-IP in BALF [7] and exhaled breath condensate (EBC) [8,9] in patients suffering from sarcoidosis have been reported. In our previous paper we reported, that higher concentrations of 8-IP in EBC are found in a more extensive disease (radiological stage III), that raises hopes, that this marker could have a prognostic value [9].

Therefore we undertook this study to evaluate the clinical value of EBC 8-IP in sarcoid patients, followed over a period of 6-12 months. We were especially interested, whether high initial EBC 8-IP concentrations predispose to more severe disease, low initial concentrations increase a chance of early remission, and whether remissions are connected with the decrease of EBC 8-IP concentrations.

## Methods

### Study group

40 caucasian patients (23 women, age 39 ± 11) with sarcoidosis were included. In all patients the diagnosis was proved by histo-pathological examination, except those with the stage I/II disease with acute onset and typical radiological and BAL features [1]. Four patients had been treated in the past, but all were treatment-naïve for at least 12 months at the time of initial evaluation. They were non-smokers, non-atopic, and had no significant co-morbidities. Respiratory infection in the last 4 weeks was an exclusion criterion. Extrapulmonary sarcoidosis was suspected or proven in 14 patients (enlarged spleen in USG - 7, skin changes other than erythema nodosum - 6, impaired liver function tests - 2, positive liver biopsy - 1, enlarged abdominal lymphnodes - 1, facial nerve palsy - 1). Although extrapulmonary involvement was not a subject of our study, regression of extrapulmonary signs present at V1 was a condition to qualify a patient as healthy at V2 (complete remission). Characteristics of the study group is presented in Table 1. Four patients from the present study group participated in the study published previously (10%) [9].

**Table 1:** Radiological stages, lung function tests parameters, frequency of patients with Löfgren syndrome, abnormal laboratory results, % of patients on treatment and disease duration in patients at initial evaluation (V1) and follow-up (V2) visits.

Feature	Visit 1	Visit 2
Stage I	23 (57.5%)	13 (-10 with CR)
Stage II	9 (22.5%)	8 (-1 with CR)
Stage III	8 (20%)	8 (no CR)
FEV_1 _(% predicted)	92.6 ± 3.2	93.8 ± 3.1
FVC (% predicted)	97.9 ± 3.0	99.7 ± 2.9
FEV_1_/FVC (%)	0.81 ± 0.02	0.80 ± 0.02
DLCOc (% predicted)	87.3 ± 3.4	87.7 ± 3.7
LS present and past	20 (50%)	-
LS present	15 (37.5%)	0
SACE > 68 IU/L	11 (27.5%)	5 (12.5%)
S-Ca^2+ ^> 2.62 mmol/L	1 (2.5%)	2 (5%)
U-Ca^2+ ^> 7.5 mmol/24 h	3 (7.5%)	5 (12.5%)
CRP > 5 mg/L	15 (37.5%)	5 (12.5%)
On treatment	0	8 (20%)
T from first symptoms to V1 (weeks, median: 25-75 percentile)	10 [4-60]	-

CR – complete remission, DLCOc – diffusion capacity for CO corrected for hemoglobin, FEV_1_ #8211; forced expiratory volume in 1^st ^second of expiration, FVC – forced vital capacity, LS – Löfgren syndrome, S – serum, SACE – serum angiotensin converting enzyme, T – time, U – urine.

### Control group

34 healthy never smokers (19 women, age 45 ± 10), members of a hospital staff, free of respiratory infection in the last 4 weeks.

### Ethics

The study was approved by Ethical Committee at Medical University of Lodz (consent N° RNN/99/08/KE) and all patients signed informed consent.

### Exhaled breath condensate collection

EBC was collected using a condensing device (Ecoscreen, Jaeger, Germany). Patients were asked to breath out spontaneously through a mouthpiece equipped with a saliva trap for 10 min. The respiratory rate ranged from 15 to 20 breaths/min. All subjects wore a nose-clip and rinsed their mouths with distilled water just before and after the 7^th ^min of the condensing process, in order to reduce nasal contamination. Samples were stored at -80°C until measurements. The collection of EBC was performed following available recommendations [10].

### Measurement of 8-isoprostane

Concentrations of 8-isoprostane in EBC were measured by a specific enzyme immonoassay (EIA) kit (Cayman Chemical, Ann Arbour, MI) as previously described [9]. The detection limit was 5 pg/ml. Levels of measured mediator below the detection limit were arbitrarily assumed to be half of the detection limit value. All samples were measured in duplicate. The intra-assay reproducibility (coefficient of variation, CV) calculated from all measurements was 13.2%. The inter-assay reproducibility was estimated on the basis of repeated measurements in 17 control subjects (samples taken over a 4 week interval). The CV was 19.0%. In 2 subjects (11.8%) the one-grade change between 8-IP concentration was found between ranges (from <5 to 5-20 pg/mL).

### Other procedures at V1 and V2

All patients had postero-anterior and lateral chest X-ray (CXR) and high resolution computed tomography (HRCT). Lung function tests were performed according to ERS/ATS standards [11,12]. FEV_1_, FVC, FEV_1_/FVC and lung diffusion capacity, corrected for haemoglobin (DLCOc) was measured. Bronchoscopy with bronchoalveolar lavage (BAL) was performed only at V1, according to British Thoracic Society Guidelines [13]. BAL was collected and worked out as described previously [9]. Blood was collected for serum angiotensin converting enzyme (SACE), CRP and calcium, 24 hrs urine was collected for calcium loss.

### Definitions of disease progression and activity

For the purpose of this study, to define evolution of the disease at visit 2 we used a simple scoring system (Table 2). According to the number of points at the final visit, the patient was put into one of the following groups:

**Table 2:** Estimation of clinical status of sarcoidosis at follow-up visit (V2).

V2 examinations	Score
Radiology (CXR)	0. normal
	1. abnormal, improved to V1
	2. abnormal, no change to V1
	3. worse than V1

LFT (FEV_1_, FVC, FEV_1_/FVC, DLCOc)	0. FEV_1_/FVC>70%, other parameters ≥ 80% predicted
	1. FEV_1_/FVC≤ 70%, at least one of the other < 80% predicted

Laboratory (SACE, CRP, S-Ca^2+^, U-Ca^2+^)	0. all within NL
	1. at least one out of NL

Lofgren syndrome (EN, elevated BT, arthritis, oedema)	0. absent
	1. present

General symptoms (fatigue, weight loss, sweating, arthralgia, myalgia etc)	0. absent
	1. present

Respiratory symptoms (cough, breathlessness, exercise intolerance etc)	0. absent
	1. present

Extrapulmonary signs and symptoms	0. absent
	1. present

Only patients with the sum of points at V2 = 0 were qualified as having complete remission. BT -- body temperature, DLCOc -- diffusion capacity for CO corrected for hemoglobin, EN -- erythema nodosum, FEV_1 _-- forced expiratory volume in 1^st ^second of expiration, FVC -- forced vital capacity, LFT - lung function tests, NL -- normal; limits, S -- serum, SACE -- serum angiotensin converting enzyme, T -- time, U -- urine.

**Complete remission **- complete regression of radiological findings and no symptoms, laboratory results normal, no signs of other extrapulmonary involvement (score at V2 = 0).

**Partial remission **- radiological improvement but chest X-ray still abnormal, or complete regression of radiological findings but at least one laboratory abnormality, or any general or respiratory symptom consistent with clinical picture of sarcoidosis present, or persistence of any other signs of pulmonary and extrapulmonary sarcoidosis (score at V2 ≥ 1, but decreased ≥ 2 points compared to V1).

**Stabilization **- no change in radiological picture and lung function tests results, no progression in clinical symptoms and new laboratory abnormalities, no new extrapulmonary manifestations (score at V2 ± 1 point compared to V1).

**Progression **- worsening of radiological findings and/or lung function tests results, new pulmonary, general and extrapulmonary symptoms (score at V2 increased ≥ 2 points). This is only theoretical, as there were no patients with progression in our study group. For statistical analysis only two groups were considered: complete remission (CR) and no complete remission (no-CR, including partial remission and stabilization).

### Statistical analysis

Data were shown as mean ± standard error of means (SEM), with exception of age (mean± standard deviation). Kolmogorow-Smirnoff test was used to assess normality. Median with 25 and 75 percentile was provided, for not normally distributed data. Unpaired T-test (for normally distributed data) or Mann-Whitney test (for non-parametric data) was used to compare sarcoidosis with the controls. Paired T-test was used for calculation of differences between V1 and V2 results of EBC 8-IP in treated patients and untreated patients with spontaneous remission. When more than 2 groups were compared with, one-way ANOVA and Bonferroni post-test (for data with Gaussian distribution) or Kruskall-Wallis followed by Dunn's Multiple Comparison Test (for data without normal distribution) were used. Relative risk (RR) as a ratio of expected to unexpected risks was calculated. The differences in frequency distribution between subgroups (remission vs no remission when 8-IP <5 or >20 pg/mL) were estimated by χ^2 ^and Fischer tests. Spearman test was applied to assess correlations. The p value ≤ 0.05 was assumed as statistically significant.

## Results

**Figure 1:**
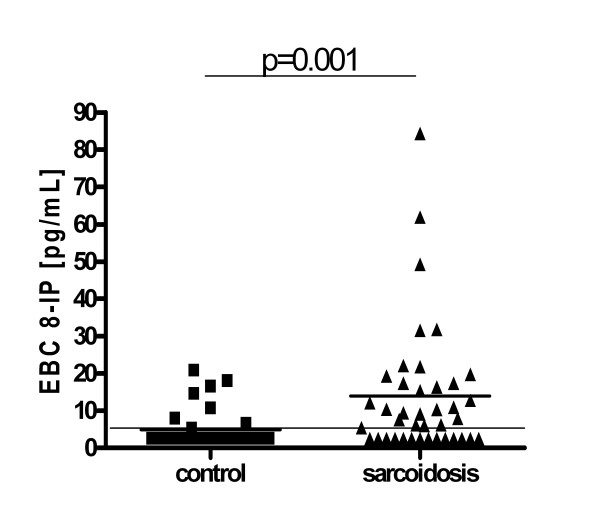
**Comparison of exhaled breath condensate (EBC) 8-isoprostane (8-IP) concentrations in healthy controls and all sarcoidosis patients**.

**Figure 2:**
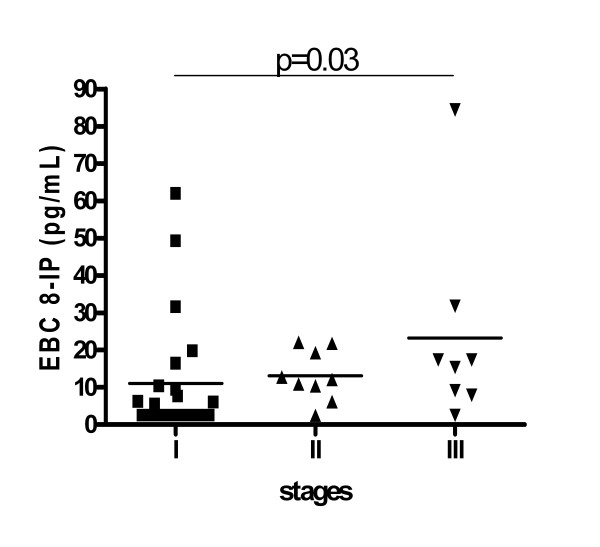
**Comparison of exhaled breath condensate (EBC) 8-isoprostane (8-IP) concentrations in sarcoidosis patients divided according to radiological stages**. There was a significant difference between stages estimated by Kruskal-Wallis test (p = 0.03). The Dunn's Multiple Comparison post-test showed the significant difference between stage I and III (p < 0.05). There were no differences between stage I and II and stage II and III (p > 0.05).

EBC 8-IP concentrations were higher in sarcoidosis (median; 25-75 percentile): 8.50; 2.50-17.40 vs 2.50; 2.50-3.90, p = 0.001. In 14 out of 40 patients (35%) and in 26 out of 34 healthy controls (76%) 8-isoprostane concentrations were below detection limit (Figure 1). Concentrations were the highest in stage III (p = 0.03 vs stage I), and the proportion of readings below detection limit were: 12/23 (52%), 1/9 (11%) and 1/8 (12.5%) in stages I, II and III, respectively (Figure 2).

Relative risk (RR) of persistence of sarcoidosis in 6-12 months of follow-up when initial 8-IP was above 20 pg/mL was 1.04 (95%CI = 0.21-4.19, p = 0.94). The chance of early remission (RR) when 8-IP was below detection limit was 3.33 (95%CI = 1.20-5.78, p = 0.03). We found a significant relationship between low 8-IP concentrations (below detection limit) and frequency of complete remission (p = 0.04). See contingency table for detailed results (Table 3).

**Table 3:** Contingency table.

		EBC 8-IP [pg/mL]			
			
		< 5	5-20	> 20	Total
CR (+)	No of patients	7	2	2	11
	% with CR	63.6	18.2	18.2	100
	% with EBC 8-IP level	50.0	10.5	28.6	27.5
	χ^2^	p = 0.04			

CR (-)	No of patients	7	17	5	29
	% with CR	24.1	58.6	17.2	100
	% with EBC 8-IP level	50.0	89.5	71.4	72.5
	χ^2^			NS	

Total	No of patients	14	19	7	40
	% with CR	35.0	47.5	17.5	100
	% with EBC 8-IP level	100	100	100	100

**Figure 3:**
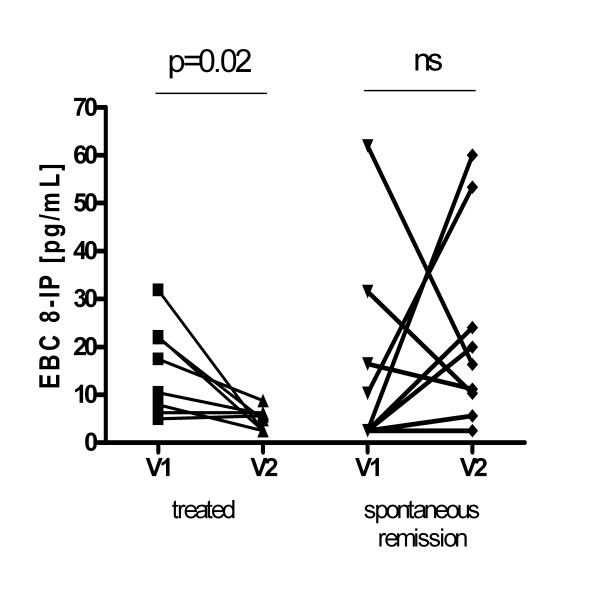
**Changes of exhaled breath condensate (EBC) 8-isoprostane (8-IP) concentrations between visit 1 -- first assessment (V1) and visit 2 -- follow up (V2) in treated patients and those who experienced spontaneous complete remission**.

A significant decrease of 8-isoprostane concentration at V2 was observed only in patients, who received systemic steroids (p = 0.02) (Figure 3), but was not observed in other patients, especially those, who experienced spontaneous complete remission. When treated patients were excluded from analysis, there were no significant differences in V1:V2 ratio between prognostic groups (CR vs no-CR).

There was no significant correlation between 8-IP or V1:V2 8-IP ratio and BAL cells, laboratory parameters of activity or duration of disease.

Table 4 shows data of BAL cellularity, lung function test parameters, laboratory parameters, time from onset of disease to V1 and frequency of Löfgren syndrome in subgroups divided according to radiological stage.

**Table 4:** BAL cytological pattern, lung function tests parameters, laboratory results, % of patients on treatment and time from first symptoms to visit 1 in sarcoidosis patients divided according to radiological stage.

	Stage I	Stage II	Stage III	Difference
BAL lymphocytes (%)	29.6 ± 3.5	34.9 ± 7.3	32.8 ± 7.0	NS
BAL lymphocytes (× 10^4^/mL)	5.03 ± 1.08	8.5 ± 2.7	10.98 ± 4.15	NS
BAL neutrophils (%)	1 ± 0.33 (1) [0-1]	1.13 ± 0.4 (1) [0-2]	0.33 ± 0.21 (0) [0-5]	NS
BAL neutrophils (× 10^4^/mL)	0.15 ± 0.06	0.22 ± 0.08	0.23 ± 0.22	NS
BAL eosinophils (%)	1.17 ± 0.25 (1) [0.5-1.5]	0.75 ± 0.25 (1) [0-1]	0.72 ± 0.32 (1) [0-5]	NS
BAL eosinophils (× 10^4^/mL)	0.14 ± 0.03	0.16 ± 0.25	0.72 ± 0.32	p ≤ 0.01: I vs III p ≤ 0.05: II vs III
FEV_1 _(% predicted)	99.4 ± 3.5	81.1 ± 7.5	87.8 ± 7.0	p ≤ 0.05: I vs II
FVC (% predicted)	104.7 ± 3.5	84.8 ± 7.0	94.9 ± 4.0	p ≤ 0.05: I vs II
FEV_1_/FVC (%)	0.83 ± 0.02	0.78 ± 0.04	0.77 ± 0.04	NS
DLCOc (% predicted)	96.8 ± 4.4	77.4 ± 6.0	77.1 ± 6.3	p ≤ 0.05: I vs II&III
SACE (IU/L)	65.8 ± 8.6	98.8 ± 30.3	45.8 ± 12.8	NS
S-Ca^2+^(mmol/L)	2.46 ± 0.02	2.44 ± 0.08	2.50 ± 0.02	NS
U-Ca^2+^(mmol/24 h)	5.25 ± 0.48	5.15 ± 0.94	4.52 ± 0.83	NS
CRP (mg/L)	7.30 ± 2.27 (5.0) [1.5-7.1]	19.85 ± 8.77 (9.4) [4.6-25.3]	4.40 ± 1.71 (2.5) [0.7-6.7]	NS
T from onset to V1 (weeks)	38 ± 25 (8) [4-24]	107 ± 50 (8) [4-238]	149 ± 55 (88) [18-289]	p ≤ 0.05: I vs III
LS	15/23	5/9	0/8	-

DLCOc -- diffusion capacity for CO corrected for hemoglobin, FEV_1 _-- forced expiratory volume in 1^st ^second of expiration, FVC -- forced vital capacity,

LS -- Löfgren syndrome, S -- serum, SACE -- serum angiotensin converting enzyme, T -- time, U -- urine, V1 -- visit 1.

## Discussion

This is the first study analyzing possible prognostic value of exhaled breath 8-isoprostanes in sarcoidosis. We confirmed earlier results, showing higher EBC 8-isoprostane concentrations, especially in patients with more advanced pulmonary changes. In this follow-up study (albeit short) we have also shown, that low 8-isoprostane (rather than high levels) may describe future prognosis, literally that patients with values below detection limit are more likely to recover spontaneously in a short period of time.

Sarcoidosis is usually a self-limiting disease, which lasts 12-36 months in 50% of patients, in the majority of cases disappears with no or minimal consequences within 5 years, at the latest [1]. Remissions within 6 months are not uncommon, especially in stage I and with those with Löfgren syndrome (LS) [1]. In our findings complete early remissions occurred in 11 out of all (40) sarcoid patients (10 were diagnosed as stage I, 1 as stage II, 8 presented LS). Therefore, we decided to analyze the rate of early remissions (6-12 months) in the context of 8-IP concentrations in EBC.

### Do high initial EBC 8-isoprostane concentrations predispose to more severe disease

Initial concentrations of 8-IP were significantly higher in patients with more advanced disease (radiological stage 3). Undetectable levels of EBC 8-IP were much less frequent in stage II and III compared to controls and patients within stage I. However, patients with the highest levels (above the highest concentration in a control group) do not show significantly increased risk of disease persistence after few months of observation. Due to a short period of observation, the preliminary character of these results should be taken into consideration. We know, that many patients experience complete remission later than after a year. In this study these patients could not have been identified. It means, that final results should be evaluated in the long-term follow-up study after at least a 2 year period, or even later.

### Do low initial concentrations increase a chance of early remission?

Our results show, that the chance of early remission is > 3 times higher in patients, who had concentrations of 8-IP in EBC below the detection limit. It is intriguing however, that some of these patients show-up with elevated EBC 8-IP after several months, regardless of the absence of clinical signs of the disease. This again, will deserve a long-term study, whether these patients are in danger of future relapse.

The value of our results was diminished by the fact that many sarcoid patients have low levels of EBC 8-IP. On the other hand, there is a substantial overlapping of sarcoidosis and control group results, therefore it is impossible to set up a clear cut-off level for "abnormal" results. In addition, although a large percentage of patients with stage I do recover early, we know from other studies and from everyday practice, that some patients experience relapses, usually within 2 years after remission. This can be evaluated in a long-term follow-up study.

### Do remissions are connected with the decrease of EBC 8-isoprostane concentrations?

We found, that only patients who received treatment between V1 and V2 (and who were on treatment at V2) have significantly reduced EBC 8-IP. In all, partial remissions were observed. This observation is especially striking in contrast with the lack of such an effect on the rest of patients, especially in those who experienced complete remission (in 5 out of 11 an increase was recorded). It suggests only unspecific role of systemic steroids, unrelated to the mere regression of granulomatous inflammation. *Antczak et al*. found decreased EBC 8-IP concentrations in inhaled steroid-treated patients with aspirin-sensitive and aspirin-insensitive asthma, comparing to steroid-naïve patients [14]. Other authors do not confirm that inhaled steroids may decrease EBC 8-IP [15], however administration of oral steroids due to asthma exacerbation in children resulted in a significant decrease of EBC 8-IP [16].

There is no data in the literature on the possible influence of steroid treatment on EBC 8-IP in sarcoidosis and other interstitial lung disease.

On the basis of our results it is difficult to explain why some patients with clinical remission have high concentrations of 8-IP in exhaled breath at final evaluation. The influence of other clinical states should be considered. Although infection and allergy were exclusion critera, we were not able to exclude subclinical states, which could have influenced the measurements. In some patients bronchial hyperreactivity may evolve [17], which would give another possible explanation for these outstanding results. Finally, we can not exclude, that in some of these subjects the disease is still active or will result in relapses.

### Oxidative stress in the pathogenesis of sarcoidosis

Our results provide an argument for the possible role of oxidative stress and lipid peroxidation in sarcoidosis. Production of superoxide anion by BAL cells is increased proportionately to the grade of lymphocytic inflammation [18]. Oxidative stress may be involved even in the very early stages of granuloma formation, as oxidants may stimulate macrophages to the production of TNF-α and other cytokines indispensable for granuloma formation. TNF-α may be detectable in EBC, however the concentrations were not elevated in sarcoidosis [19]. Although, we have not measured TNF-α in our patient's EBC, such data would be interesting in the context of the possible application of anti-TNF agents in the treatment of refractory sarcoidosis [20]. Elevated 8-IP concentrations in EBC may be related to the destruction of cellular membranes, but further studies are required to evaluate whether patients with high EBC 8-IP have a higher risk of lung fibrosis.

## Conclusions

This short-term follow-up study does not provide an evidence for negative prognostic value of high levels of EBC 8-IP. A long-term follow up study, involving more numerous and clinically heterogenous group of patients is necessary for conclusive evaluation. However, our data shows, that low concentrations of 8-IP in EBC may predispose to early remission. Complete remissions are not connected with a consistent decrease of EBC 8-IP. Only in patients treated with steroids, regardless the remission was achieved, the decrease of EBC 8-IP was noticed.

## Abbreviations

BAL: bronchoalveolar lavage; CR: complete remission; DLCO: diffusion capacity for carbon monoxide; DLCOc: DLCO corrected for hemoglobin concentration; EBC: exhaled breath condensate; EIA: enzyme immunoassay; FEV_1_: volume in 1^st ^second of forced expiration; FVC: forced vital capacity; 8-IP: 8-isoprostane; LS: Löfgren syndrome; RR: relative risk; SACE: serum angiotensin converting enzyme

## Competing interests

The authors declare that they have no competing interests.

## Authors' contributions

WJP was responsible for study design, data collection, statistical analysis, data interpretation, manuscript preparation, literature search and funds collection. ZK was responsible for laboratory work and data collection. AA was responsible for review and correction of a manuscript and funds collection. JM was responsible for data collection. PG was responsible for review and correction of a manuscript and funds collection. All authors read and approved the final manuscript.

## Pre-publication history

The pre-publication history for this paper can be accessed here:

http://www.biomedcentral.com/1471-2466/10/23/prepub
